# Impact of Temperature on Survival Rate, Fecundity, and Feeding Behavior of Two Aphids, *Aphis gossypii* and *Acyrthosiphon gossypii*, When Reared on Cotton

**DOI:** 10.3390/insects12060565

**Published:** 2021-06-21

**Authors:** Jinping Liu, Chen Wang, Nicolas Desneux, Yanhui Lu

**Affiliations:** 1 State Key Laboratory for Biology of Plant Diseases and Insect Pests, Institute of Plant Protection, Chinese Academy of Agricultural Sciences, Beijing 100193, China; jinping_liu21@163.com (J.L.); chenwang_wc@163.com (C.W.); 2 Université Côte d’Azur, INRAE, CNRS, UMR ISA, 06000 Nice, France; nicolas.desneux@sophia.inra.fr

**Keywords:** climate change, high temperature, cotton aphid, electrical penetration graph, adaptability

## Abstract

**Simple Summary:**

Understanding the effects of high temperature on pest insects is important in predicting their population dynamics. *Aphis gossypii* (Glover) and *Acyrthosiphon gossypii* (Mordviiko) are the two most destructive aphids on cotton in Xinjiang Province, China. This study examined the effect of different temperatures (29 °C, 32 °C, and 35 °C) on the adult survival rate, fecundity, and feeding behavior of *Ap. gossypii* and *Ac. gossypii*. Our results showed that the adverse effects of high temperatures (32 °C, 35 °C) on aphid adult survival and fecundity were greater for *Ac. gossypii* than *Ap. gossypii*. Feeding data showed that *Ac. gossypii* spent more time feeding on xylem than phloem under all temperature treatments, which contrasted with *Ap. gossypii*. The time of phloem ingestion by *Ap. gossypii* at 32 °C was significantly higher than at 29 °C, while for *Ac. gossypii*, this value significantly decreased when temperature increased. These feeding patterns indicate that *Ac. gossypii* obtains less nutrition from phloem in support of its development and fecundity. Data generated in this study will serve as the basis for predicting the effect of increased temperature on these two cotton aphids.

**Abstract:**

Aphid performance is sensitive to temperature changes. Previous studies found that *Acyrthosiphon gossypii* (Mordviiko) was more sensitive to high temperature than *Aphis gossypii* (Glover). However, the effects of high temperatures on the survival, fecundity, and feeding behavior of these two aphid adults are not clear. This study examined the effect of different temperatures (29 °C, 32 °C, and 35 °C) on the adult survival rate, fecundity, and feeding behavior of these two aphid species. Our results showed that the adverse effects of high temperatures (32 °C and 35 °C) on aphid adult survival and fecundity were greater for *Ac. gossypii* than *Ap. gossypii*. The electrical penetration graph (EPG) data showed that *Ac. gossypii* spent more time feeding on xylem than phloem under all temperature treatments, which contrasted with *Ap. gossypii*. The time of phloem ingestion by *Ap. gossypii* at 32 °C was significantly higher than at 29 °C, while for *Ac. gossypii*, this value significantly decreased when temperature increased. These feeding patterns indicate that *Ac. gossypii* obtains less nutrition from phloem in support of its development and fecundity. Data generated in this study will serve as the basis for predicting the effects of increased temperature on these two cotton aphids.

## 1. Introduction

As one of most important current environmental issues, global warming is of wide concern. The mean global temperature increased by 0.72 °C from 1880 to 2012, and it is predicted to rise an additional 1.1 °C to 6.4 °C by the end of the 21st century [1,2]. Such climate changes present a major challenge for poikilotherms such as insect herbivores [3]. Most previously published studies have found that increases in atmospheric temperature directly affect life cycles, phenology, behavior, and distribution of insect herbivores, or indirectly affect insects through other factors that respond to temperature-induced changes [4,5,6,7]. For example, even moderate increases of ambient temperature can enhance the population growth rates of some insect herbivores, resulting in more generations per year [8,9]. A higher temperature in spring or winter can modify the normal developmental time and the overwintering of insects [10]. An increase in night-time temperature (>20 °C) can reduce the survival and fecundity of English grain aphid, *Sitobion avenae* (Fabricius) [11]. In addition, *S. avenae* avoids heat stress by dropping off its host plant [6], which interrupts feeding. The field occurrence and period of migration of *Aphis gossypii* (Glover) have both increased historically along with climate warming based on a period of about 60 years of literature data [12]. Species may respond differently to rising temperatures, and the degree of change species undergo may depend on their individual ability to adapt to high temperature, whether through rapid evolution or high plasticity [13]. Changes in insect herbivores’ performance under climate warming conditions may affect agricultural production strategies and pest control. Therefore, it is of great importance to study the effects of temperature on insects as a basis for predicting the future status of insect pests.

Aphids have a small body size and their development, survival, and reproduction are sensitive to changes in ambient temperature [11,14]. *Aphis gossypii* is a highly polyphagous pest infesting nearly 100 crop species throughout the world [15]. The crops damaged by *Ap. gossypii* include cotton, cucurbits, citrus, coffee, cocoa, aubergine, peppers, potato, and okra, as well as many ornamental plants. *Acyrthosiphon gossypii* (Mordviiko) has a narrower range of distribution in China compared with *Ap. Gossypii*, being found only in Xinjiang and Gansu provinces. *Acyrthosiphon gossypii* damages legumes and cotton [16]. In Xinjiang Province, both *Ap. gossypii* and *Ac. gossypii* co-occur as important pests of cotton [17]. However, *Ap. gossypii* may occur in the seedling, flowering, and boll stage of cotton growth, while *Ac. gossippii* generally occurs in the seedling stage [18].

Previous studies indicate that *Ap. gossypii* and *Ac. gossypii* respond differently to high temperature [17]. For *Ap. gossypii*, Kersting et al. [19] reported that the net reproduction rate (*R*_0_) was 37.9 at a constant 30 °C, while 35 °C was lethal to *Ap. gossypii* nymphs reared on cotton. However, some studies have demonstrated that the first instars of nymph of *Ap. gossypii* reared on cotton and cucumber can successfully survive to the adult stage and produce progeny at a constant 35 °C [20,21,22,23]. The host plant species and aphid genotype may explain this difference in the value of the upper lethal temperature for *Ap. gossypii*.

In comparison, *Ac. gossypii* nymphs can successfully survive to adult stage at constant 30 °C, but female adults fail to produce offspring [18]. Although earlier studies have tested the impact of constant temperatures on the developmental time and fecundity of *Ap. gossypii* and *Ac. gossypii* reared on cotton [16,19,20,23], most of these studies started with first instar nymphs in each temperature. These studies show that temperature may affect nymphal instars differently [24], and clearly the adult stage, not considered in many of these earlier studies, plays important population roles in the areas of reproduction and dispersal. Therefore, the impact of increasing temperature on *Ap. gossypii* and *Ac. gossypii* needs to be studied more holistically by including adult responses, together with that of each separate instar.

The feeding behavior of aphids can be affected by the nutritional quality of the phloem sap of the plants on which they feed. Phloem quality itself can be affected by climate change [25,26,27,28]. Jiang et al. [29] found that the time of stylet penetration to phloem position and the mean frequency of xylem phase of *Ap. gossypii* were significantly reduced under elevated CO_2_ because of increased soluble proteins in the cotton leaves. The feeding efficiency of *Myzus persicae* (Sulzer) increased under elevated CO_2_ owing to up-regulation of mitogen-activated protein kinases (MPK4) in the host plant [27]. The xylem absorption time of *Acyrthosiphon pisum* (Harris) was increased to deal with high osmotic pressure of phloem sap under drought stress [25]. Because aphids vector many plant viruses [30], the details of aphid feeding behavior are important because any changes may affect the virus’ transmission rate [31]. Therefore, studies of feeding behavior of aphids under elevated temperature are helpful in predicting changes in the transmissibility of plant viruses. Currently, such information regarding the impact of temperature on the feeding behavior patterns of *Ap. gossypii* and *Ac. gossypii* is not available. It is not known whether or not the impacts of high temperatures on the feeding behaviors of these two long-coexisting aphids will be similar.

The electrical penetration graph (EPG) technique has been used to monitor the probing behavior and location of stylet tips in the host tissue of the sucking insect for a long time [32]; the results can help us to identify the host preference [33,34], host resistance [35], and position of host resistance [36] to insects. Here, our specific goals were (i) to compare the survival rates, longevity, and fecundity of adult females of *Ap. gossypii* and *Ac. gossypii* at different temperatures (29 °C, 32 °C, and 35 °C) and (ii) to use the electrical penetration graph (EPG) technique to investigate the feeding behaviors of *Ap. gossypii* and *Ac. gossypii* among these temperatures. These data will provide a necessary basis for predicting the effects of increased temperature on the performances of these two cotton aphids.

## 2. Materials and Methods

### 2.1. Aphids Sources and Host Plants

**Figure 1:**
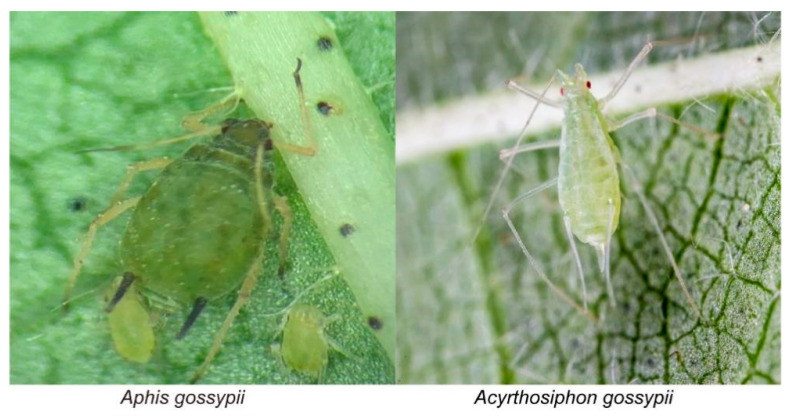
Adult of Aphis gossypii and Acyrthosiphon gossypii.

*Aphis gossypii* and *Ac. gossypii* were collected from cotton fields on Korla Experimental Station, Chinese Academy of Agricultural Sciences (CAAS; 41.45° N, 85.48° E) (Korla, Xinjiang Province) on 21 June 2019. We identified the two aphid species based on the morphological characteristics described in Li [37]. The adults are shown in Figure 1. The cotton field was maintained without any pesticide before aphids were collected. Aphids were reared on young cotton leaves in a controlled climatic chamber at 29 ± 1 °C, 50 ± 5% RH, and 16:8 (L/D) h photoperiod at the Langfang Experimental Station, Chinese Academy of Agricultural Sciences (CAAS; 39.53° N, 116.70° E) (Langfang, Hebei Province). The *Gossypium hirsutum* L. cultivar Zhongmian 49 was used in this study. Cotton plants were grown from seeds in plastic cups (top diameter, 120 mm; bottom diameter, 90 mm; height, 100 mm) containing peat, vermiculite, and field soil (volume ratio: 6:1:1) in a greenhouse at 28–30 °C, 50 ± 5% RH, and 16:8 (L/D) h photoperiod. Cotton plants were used for the feeding behavior experiment at the 4- to 5-leaf stage.

### 2.2. Tested Temperatures

The values of the tested temperatures were determined based on previous studies [2,16,19,20] and our preliminary experiment. Here, we chose three temperatures: 29 °C (optimal temperature, as control), 32 °C (moderately increased temperature), and 35 °C (severely high temperature).

### 2.3. Effects of Temperature on Survival and Fecundity of Adults

Wild aphid population was reared in indoor chambers for one generation. One-day-old apterous adults from the first generation in indoor culture were randomly collected with a fine brush and transferred onto the lower surface of an excised leaf disk of cotton that was placed in small plastic box (diameter: 60 mm, high: 50 mm) on a layer of 1% agarose. To avoid aphid escape, the box was covered with 100 mesh gauze. Adults were allowed to produce progeny at 29 °C, and then transferred onto a new leaf disk after 24 h. Newly produced nymphs were left in place and reared on their natal leaf. These nymphs were subsequently transferred onto new cotton leaf disks every 2–3 days. When nymphs had molted four times and developed to the adult stage, a group of 100 one-day-old adults (<12 h old) were selected randomly and placed in chambers under a constant temperature (29 °C, 32 °C, or 35 °C), with 50 ± 5% RH, and 16:8 (L/D) h photoperiod. Ten adults were placed in each test plastic box (as mentioned above); so each temperature treatment had ten boxes. A group of ten adults constituted one. Adults were checked daily to record their survival and count the newly produced nymphs, which were removed daily. The aphid adults were transferred onto new cotton leaf disks every 1–2 days. Observations continued until all adults died.

### 2.4. Electrical Recording of Aphid Feeding-Behaviors

A Giga-8 direct current electrical penetration graph (DC-EPG) amplifier system with a 1 Giga Ω input resistance and an input bias current <1 pA in a Faraday cage (manufactured by Wageningen University, Wageningen, The Netherlands) was used to record the feeding behaviors of aphid adults on cotton in this experiment [38]. The structural design of this system is shown in Supplementary Figure S1. Newly emerged apterous adult aphids (<12 h old), reared on cotton leaf at 29 °C, were randomly selected and were subjected to a 1 h starvation period before recording their behaviors at the control temperature condition. A gold wire (12.5 μm in dia, 2 cm long) was attached on the dorsum of the aphid with water soluble conductive silver glue. The gold wire was glued to copper wire (2 mm dia × 2 cm length) attached to a brass pin that was inserted into the EPG probe as the insect electrode. The aphids, which were glued to gold wire, were transferred into the tested room at a fixed temperature condition that had been set in advance (29 °C, 32 °C, 35 °C). The cotton plants grown at the control temperature condition (28–30 °C) were simultaneously transferred into the tested room. The temperature was checked every two hours to maintain the fixed temperature during the whole experiment. A copper wire (2 mm dia × 10 cm length) was inserted into the soil of the pot as the plant electrode. As the aphid’s stylets penetrated the plant, the circuit was completed, and a fluctuating voltage was amplified with the Giga-8 DC amplifier between ±5 V at the amplifier output, and recordings were made on Stylet + d software. The EPG device, in an electrically grounded Faraday cage, was placed in a quiet room to avoid external noise. Aphids were gently placed onto the lower surface of a single cotton leave (on a plant at four-leaf stage) and this test leaf was tied to a stick to hold it steady. Using an eight-channel amplifier, we simultaneously recorded eight individual aphids on separate plants for 8 h. The two species, *Ap. gossypii* and *Ac. gossypii*, were recorded simultaneously and each species was randomly assigned to four of the available channels. New cotton plants and new aphids were used for each recording session. The EPG analysis was based on data collected from more than 18 aphids for each treatment (species × temperature). The EPG data were annotated using the Stylet + a software (Wageningen Agricultural University, Wageningen, The Netherlands). Waveform patterns were scored and assigned to previously described categories [38]: (1) non penetration (NP), representing non-probing behavior where the stylets are not in contact with the leaf surface; (2) initiation of stylet penetration of leaf tissue (which correlates with the intercellular apoplastic stylet pathway located at the epidermis/mesophyll cell layers in which the aphid shows a cyclic activity of mechanical stylet penetration and secretion saliva (C)); (3) short bouts of intracellular penetration, which leads to a signal potential drop (Pd); (4) secretion of saliva into phloem sieve elements at the beginning of the phloem phase (E1); (5) passive phloem sap uptake from the sieve element, i.e., phloem ingestion (E2); (6) ingestion of xylem sap (G); and (7) period of mechanical difficulty in stylet penetration (F).

### 2.5. Statistical Analysis

Statistical analysis was performed with Microsoft Excel (Microsoft, Redmond, WA, USA) and SPSS v.20.0 software (IBM Corporation, Armonk, NY, USA). All data were first checked for normality and homogeneity of variance and were transformed when they did not fit a normal distribution. The results from two independent groups were compared using a Student’s t-test. One-way analyses of variance (ANOVAs) were performed to determine the effects of temperature on feeding behavior, and the Bonferroni method was used to adjust the *p*-values, which were considered statistically significant between temperature treatments at *p* < 0.05. Survival statistics was calculated using the Kaplan–Meier survival curve and compared using the log-rank test. Graphs were made by GraphPad Prism 8.0 (GraphPad Software, La Jolla, CA, USA). If not otherwise stated, statistical significance was indicated as follows: *, *p* < 0.05; **, *p* < 0.01; ***, *p* < 0.001.

## 3. Results

### 3.1. Survival, Longevity, and Fecundity

**Figure 2:**
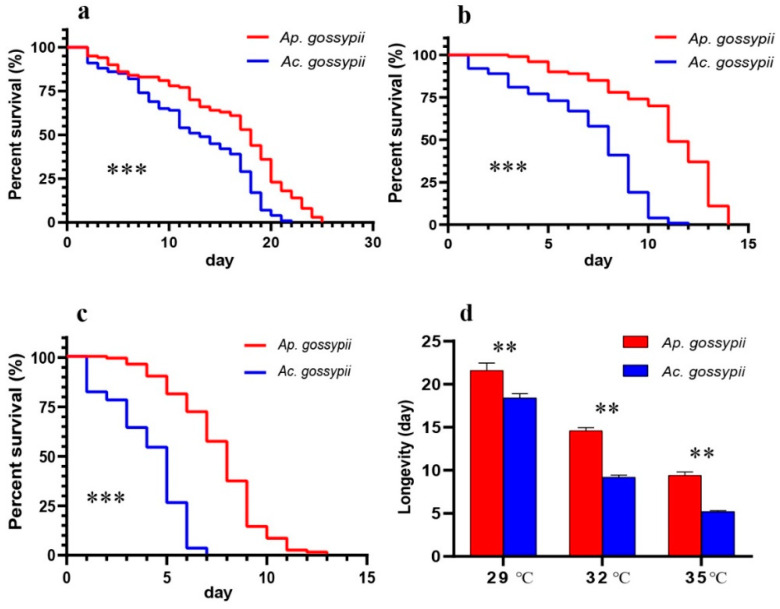
Survival curves of adults of *Aphis gossypii* and *Acyrthosiphon gossypii* at three temperatures: (**a**) 29 °C; (**b**) 32 °C; (**c**) 35 °C; and (**d**) longevity of adults of both species. Survival statistics were calculated using the Kaplan–Meier survival curve and compared using the log-rank test (individual = 100). The results from two independent groups of longevity were assessed using Student’s t-test. The error bars indicate standard error (SE). **, *p* < 0.01; ***, *p* < 0.001.

The survival rates of *Ap. gossypii* and *Ac. gossypii* were each significantly affected by high temperatures (Figure 2a–c. a: *p* < 0.0001; b: *p <* 0.0001; c: *p* < 0.0001). Both aphid species died sooner at 35 °C than at 29 °C (Figure 2a,c). At 35 °C, all *Ac. gossypii* died by day 7, while 57% of *Ap. gossypii* were still alive. At 32 °C, all *Ac. gossypii* died within 12 days, while 49% of *Ap. gossypii* survived >12 days (Figure 2b).

In general, the longevity of *Ac. gossypii* was reduced to a greater extent by high temperatures than the longevity of *Ap. gossypii* (Figure 2d). For example, an increase from 29 to 35 °C caused the longevity of *Ac. gossypii* to drop by 72%, while that of *Ap. gossypii* declined only 53%.

**Figure 3:**
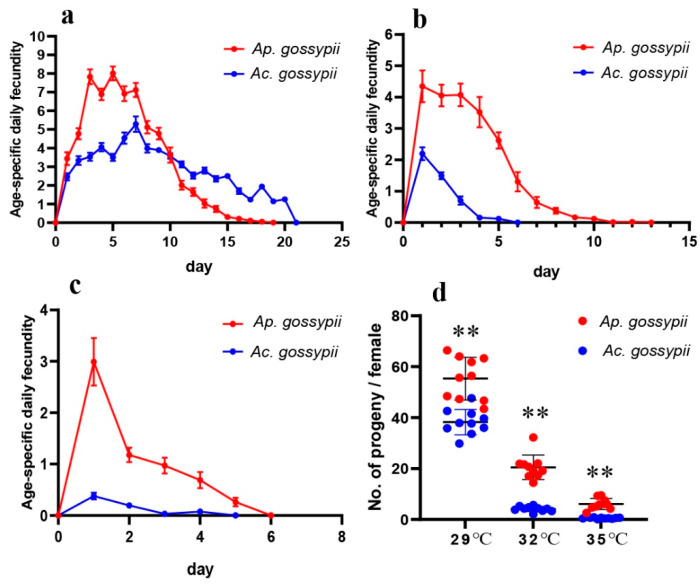
Age-stage-specific fecundity of adults of *Aphis gossypii* and *Acyrthosiphon gossypii* at different temperatures. (**a**) 29 °C; (**b**) 32 °C; (**c**) 35 °C; and (**d**) the scatter diagram of progeny per female. The results from two independent groups of fecundity were assessed using Student’s t-test. The error bars indicate standard error (SE). **, *p* < 0.01.

Age-specific fecundity of both aphids was reduced by high temperatures (Figure 3a–c), but this occurred at a lower temperature and more severely for *Ac. gossypii*. Using the maximum age-specific daily fecundity to express this effect, for the more tolerant species (*Ap. gossypii*), fecundity dropped from 8.01 to 4.34 to 2.99 as temperatures rose from 29 to 32 to 35 °C, and the peak of oviposition shifted from day 5 at the lowest temperature to day 1 at the two higher temperatures. For *Ac. gossypii*, daily fecundity was much lower that for the other aphid species at all temperatures, but daily maximum fecundity for this second species showed the same pattern of reduction, from 2.46 to 2.19 to 0.38 for 29, 32, and 35 °C, respectively, with these peaks being on day 1 for all temperatures.

### 3.2. Feeding Behavior

**Figure 4:**
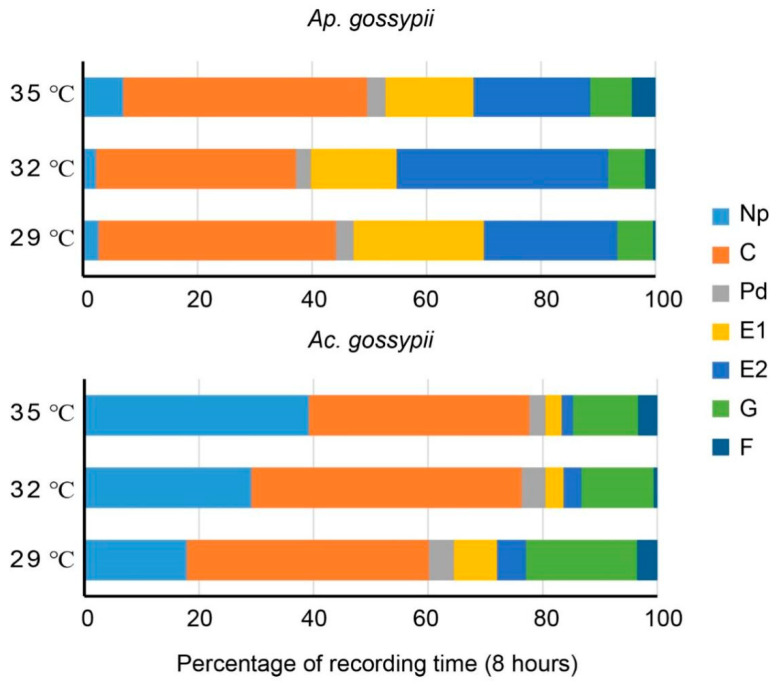
Proportional duration of various waveforms of *Aphis gossypii* and *Acyrthosiphon gossypii* behavior at different temperatures during an 8 h EPG recording. EPG waveforms are as Np = non-probing/penetration; C = pathway waveform; Pd = potential drop; E1 = salivary secretion; E2: phloem sap ingestion; G = xylem feeding; F = mechanical penetration difficulties.

Data on the non-phloem phase variables of *Ap. gossypii* and *Ac. gossypii* at different temperatures (Table 1) show that the duration of non-probe (Np) events such as walking and resting for *Ap. gossypii* was significantly longer at 35 °C than at 29 °C (*p* < 0.001) and 32 °C (*p* < 0.001). However, the time spent in Np (non-probing/penetration) by *Ac. gossypii* was significantly higher than for *Ap. gossypii* at all temperatures (29 °C: *p* < 0.001; 32 °C: *p* < 0.001; 35 °C: *p* < 0.001; t-test); moreover, the time of the np phase of *Ac. gossypii* increased with rising temperatures. The percentage of non-probing behaviors of the total recording (8 h) was 2.71%, 2.28%, and 7.03% at 29 °C, 32 °C, and 35 °C for *Ap. gossypii*, respectively, which was lower than for *Ac. gossypii* (29 °C: 17.89%; 32 °C: 29.12%; 35 °C: 39.15%) (Figure 4). The intercellular stylet pathways (C) time was lower at 32 °C for *Ap. gossypii* compared with the other two temperatures. However, no significant differences were observed in the duration of the C waves (*p* = 0.096) and potential drops (Pd) (*p* = 0.055) of *Ac. gossypii* between the three temperatures. No significant differences were observed in the duration of short potential drops (Pd) (*p* = 0.109) or times spent feeding on xylem (G) (*p* = 0.942) for *Ap. gossypii* at all temperatures. However, the number of intercellular stylet pathways event (C) and potential drops (Pd) at 35 °C was significantly higher for *Ap. gossypii* than the other two temperatures. These results show that the number (*p* = 0.043) of potential drops (Pd) for *Ac. gossypii* at 35 °C was significantly lower than at 29 °C. The duration of xylem feeding events (G) for *Ac. gossypii* at 35 °C was significantly lower than at 29 °C (*p* = 0.039).

For the phloem-feeding phase variables (Table 2), there were significant differences between *Ap. gossypii* and *Ac. gossypii*. Specifically, *Ap. gossypii* at 32 °C (*p* = 0.029) and 35 °C (*p* = 0.021) spent significantly less time on phloem salivation (E1) compared with at 29 °C. However, the phloem ingestion (E2) time of *Ap. gossypii* at 32 °C was longer than at the other two temperatures, which accounts for 37.0% of recorded time, which was higher than at 29 °C (23.4%) or 35 °C (20.4%) (Figure 4). The duration of salivary secretion events (E1) of *Ac. gossypii* at 29 °C was significantly higher than at 32 °C (*p* = 0.004) and 35 °C (*p* = 0.003). Temperature had no impact on the number of E1 or E2 events for *Ap. gossypii*. However, for *Ac. gossypii*, the numbers of E1 and E2 events were significantly affected by 35 °C compared with 29 °C. No significance was observed in the times for first salivary secretion (E1) (*p* = 0.551) or first established phloem feeding (E2) (*p* = 0.295) for *Ap. gossypii* among the three temperatures.

## 4. Discussion

This is the first study to investigate the feeding behavior of *Ap. gossypii* and *Ac. gossypii* adults, at different temperatures, using the electrical penetration graph (EPG) technique. Moreover, the effects of high temperatures on the survival rates and fecundity of adults for these two co-existing cotton aphids were determined.

There were no deaths for *Ap. gossypii* within the first 24 h under any of the temperatures examined. However, no deaths during the first 24 h for *Ac. gossypii* were observed at 29 °C, and the species’ survival rate decreased faster than that of *Ap. gossypii* with the increase of treatment time. Heat stress can also modify the age-specific daily fecundity of *Ap. gossypii* and *Ac. gossypii*. The number of offspring produced by one adult of *Ap. gossypii* was 4.35 at 32 °C in the first 24 h, which was higher than the value for this aphid when held at 29 °C (3.44) or 35 °C (2.99). However, the fecundity of *Ac. gossypii* within 24 h showed differences between 29 °C and 32 °C. The age-specific daily fecundity values for both *Ap. gossypii* and *Ac. gossypii* at 32 °C and 35 °C decreased continuously over time after day one, which contrasts results from 29 °C. Overall, the fecundity of these two aphids was eventually impaired as the time under heat stress increased. These results indicated that heat stress had a greater negative effect on the survival rate and the fecundity of *Ac. gossypii* than it did on that of *Ap. gossypii*, which is consistent with previous studies [16,20].

Our results also found that *Ac. gossypii* spent more time feeding on xylem than on phloem under all temperatures, which contrasted with the behavior of *Ap. gossypii*, possibly for two reasons. First, it is possible that these two aphids simply have different feeding strategies. It may be that *Ac. gossypii* needs more water and inorganic salt than *Ap. gossypii*, with a feeding pattern more similar to three grain aphids (*S. avenae*, *Sitobion graminum* (Rondani), and *Rhopalosiphum padi* (Linnaeus)) [39]. *Rhopalosiphum padi* spends more time feeding on xylem than the other two grain aphid species. Xylem consumption helps aphids cope with the osmotic effects associated with phloem feeding [40]. Moreover, aphids engage in more xylem feeding than phloem feeding if the plant is unacceptable [41]. The increase in xylem feeding by *Ac. gossypii* may have been to compensate for its reduced ingestion of phloem, which would be consistent with the observation that starved aphids also increase their time spent in xylem feeding [42,43]. The time of xylem feeding for *Ac. gossypii* significantly decreased at 35 °C, in contrast with *Ap. gossypii*, which indicates that the severe high temperatures have adverse effects on xylem feeding for *Ac. gossypii*.

For aphids to successfully feed on phloem sieve elements, the aphids need to secrete saliva (E1 behavior) containing Ca^2+^-scavenging proteins that help prevent phloem proteins from clogging inside sieve elements. During actual phloem feeding (E2 behavior), further saliva is added to the ingested sap, likely to prevent phloem proteins from clogging inside the capillary food canal of the aphid [42,44,45]. With the aid of these saliva-based factors, aphids can ingest phloem sap continuously for extended periods. *Aphis gossypii* fed on resistant melon plants showed longer phloem salivation periods and no sustained (>10 min) periods of phloem feeding, in contrast with feeding behavior on susceptible melon plants [46]. *Acyrthosiphon pisum* spent less time in E2 ingestion on a non-preferred host plant before acclimation when compared with a preferred host plant [47]. In the present study, a significantly lower time of E2 ingestion for *Ap. gossypii* reared on cotton plant at 35 °C compared with 32 °C indicated that *Ap. gossypii* is unable to sustain phloem feeding for a long time when exposed to severe high temperatures (35 °C). Extrapolating from the time spent by *Ac. gossypii* in our study on Np phase activities, we found that, at 35 °C, aphids needed less time to insert their stylets into the plant tissue. Moreover, we observed no sustained period of phloem ingestion (E2 > 10 min) for *Ac. gossypii* at any of the tested temperatures (Table 2), which suggests that *Ac. gossypii* was not able to sustain phloem ingestion. Data for phloem ingestion levels showed that moderate heat stress (32 °C) can improve feeding efficiency of *Ap. gossypii*, but the beneficial effects of increased temperature were limited to a range of just a few degrees and became negative above *Ap. gossypii*’s heat tolerance level (35 °C). In contrast, for *Ac. gossypii*, both 32 and 35 °C had adverse effects on phloem-feeding-related activities. This difference shows that the feeding behavior of *Ac. gossypii* is more sensitive to increasing temperature than adults of *Ap. gossypii*.

Phloem sap provides aphids with nutrients such as amino acids and sugar resources, which are needed for growth and reproduction [48,49]. In alfalfa, phloem amino acid balance affects plant resistance, which can affect *A. pisum* reproduction [50]. Pompon et al. [40] reported that the number of nymphs produced by *Macrosiphum euphorbiae* (Thomas) was negatively correlated with the proportion of time spent in xylem feeding. The lack of phloem feeding may be one of the reasons for the severely reduced fecundity of *Ac.* *gossypii* under heat stress. However, a high temperature can affect the survival rate and fecundity of insects by changing their physiological metabolic rates and modifying the spatial conformation of protein [51,52], which is a very complex process. Furthermore, different insect species show different thermal tolerances, and thus have different levels of fitness [53,54]. Previous studies reported that aphid’s intracellular *Buchnera* and facultative symbiont such as *Serratia symbiotica* play a crucial role in thermal tolerance of aphids [54,55]. Aphid’s facultative symbiont can affect aphid feeding behavior. For example, *Rhopalosiphum padi* infected with facultative symbiont, *Hamiltonella defensa*, reared on barley exhibited a twofold higher probability of sustained phloem ingestion than uninfected aphids [56]. Future study is needed to determine the shifts in obligatory, and facultative endosymbiont infections of *Ap. gossypii* and *Ac. gossypii* to explain why the fitness of these aphids was reduced under heat stress. A recent study showed that heat shock genes are involved in protein interaction and as function molecular chaperones to improve aphid heat tolerance [57]. The expression of HSP83 for *A. pisum* was reduced by RNA interference and, consequently, both the longevity and fecundity of the aphid were reduced [58]. In our study, the EPG technique was used to help us to understand the differential effects of high temperatures on probing and feeding activities of *Ap. gossypii* and *Ac. gossypii*. However, future work is needed to consider the role of phloem sap chemical composition relative to insect resistance, salivary proteins, endosymbionts, and Hsp genes to help determine the underlying mechanisms and gain a better understanding of how we can reduce thermal tolerance in pest aphids under a future warmer climate.

## 5. Conclusions

In the present study, we examined the effects of different temperatures (29, 32, and 35 °C) on adult survival, fecundity, and feeding behavior of two cotton-feeding aphid species. Our results show that the adverse effects of high temperatures on adult survival rate and fecundity were greater for *Ac. gossypii* than *Ap. gossypii*. The feeding data we collected showed that *Ac. gossypii* spent more feeding on xylem than phloem under all temperature treatments, which contrasted with *Ap. gossypii*. Moreover, xylem feeding of *Ap. gossypii* was not affected by heat stress. However, for *Ac. gossypii*, heat stress had adverse effects on both phloem ingestion and xylem feeding. Data generated in this study will serve as the basis for predicting the effects of increased temperatures on these two cotton aphids.

## Figures and Tables

**Table 1:** Comparison of electrical penetration graph (EPG) parameters of *Aphis gossypii* and *Acyrthosiphon gossypii* in the non-phloem phase under different temperatures.

EPG Parameters	*Aphis gossypii*			*Acrythosiphon gossypii*		
	29 °C (*n* = 18)	32 °C (*n* = 18)	35 °C (*n* = 26)	29 °C (*n* = 19)	32 °C (*n* = 18)	35 °C (*n* = 20)
Total duration of Np (min)	13.00 ± 1.56 bB	10.97 ± 1.27 bB	33.77 ± 2.32 aB	85.88 ± 7.49 cA	139.80 ± 10.45 bA	187.92 ± 14.34 aA
Total duration of C (min)	199.26 ± 9.96 aA	167.49 ± 8.56 bB	204.41 ± 10.55 aA	202.64 ± 13.34 aA	226.70 ± 15.01 aA	184.69 ± 12.55 aA
Total duration of Pd (min)	14.75 ± 0.82 aB	12.78 ± 0.89 aB	15.74 ± 0.96 aA	21.15 ± 2.40 aA	20.06 ± 1.89 aA	14.05 ± 1.69 aA
Total number of C	251 ± 11.37 bB	225.20 ± 11.81 bB	307.78 ± 14.15 aB	329.93 ± 20.50 aA	293.29 ± 15.15 abA	242.85 ± 13.24 bA
Total number of pd	158 ± 10.52 bB	145 ± 11.79 bB	202 ± 12.71 aB	230.4 ± 16.7 aA	258.2 ± 18.6 aA	169.92 ± 14.38 bA
Total duration of G	30.1 ± 4.56 aB	31.05 ± 5.18 aB	34.97 ± 5.77 aB	93.07 ± 10.16 aA	60.62 ± 7.87 abA	54.58 ± 8.06 bA

Means ± SE followed by the different lowercase letters indicate significant differences among the three temperature treatments within the same aphid species using the Bonferroni method at a 5% significance level, while different uppercase letters indicate significant differences between *Ap. gossypii* and *Ac. gossypii* within the same temperature treatment using Student’s t-test at a 5% significance level. EPG waveforms are Np = non-probing/penetration; C = pathway waveform; Pd = potential drop; G = xylem feeding. The numbers in parentheses are the numbers of aphids tested at each temperature treatment.

**Table 2:** Comparison of EPG parameters of *Aphis gossypii* and *Acyrthosiphon gossypii* in the phloem phase under different temperatures.

EPG Parameters	*Aphis gossypii*			*Acyrthosiphon gossypii*		
	29 °C (*n* = 18)	32 °C (*n* = 18)	35 °C (*n* = 26)	29 °C (*n* = 19)	32 °C (*n* = 18)	35 °C (*n* = 20)
Total duration of E1 (min)	108.83 ± 10.78 aA	71.71 ± 8.49 bA	73.65 ± 7.97 bA	36.10 ± 5.10 aB	14.78 ± 1.75 bB	13.36 ± 1.17 bB
Total duration of E2 (min)	112.28 ± 14.34 bA	177.49 ± 14.25 aA	97.70 ± 8.47 bA	24.17 ± 1.69 aB	15.06 ± 1.71 bB	9.37 ± 1.01 cB
Percentage of E1 + E2	0.461 ± 0.037 aA	0.519 ± 0.032 aA	0.357 ± 0.023 bA	0.125 ± 0.011 aB	0.062 ± 0.006 bB	0.047 ± 0.003 bB
Total number of single E1	4.58 ± 0.39 abB	3.42 ± 0.55 aB	5.22 ± 0.85 aA	6.61 ± 1.01 aA	6.45 ± 0.86 aA	5.92 ± 0.63 aA
Total number of E1	24.62 ± 2.17 aA	21.73 ± 2.14 aA	24.07 ± 2.43 aA	13.12 ± 1.37 aB	10.70 ± 1.15 abB	7.92 ± 0.92 bB
Total number of E2	13.61 ± 1.85 aA	12.46 ± 1.68 aA	12.69 ± 1.75 aA	4.54 ± 0.53 aB	3.17 ± 0.36 abB	2.50 ± 0.42 bB
Time to first E1 (min)	64.12 ± 13.82 aB	67.23 ± 16.02 aB	49.08 ± 8.68 aB	93.27 ± 16.12 aA	109.20 ± 14.2 aA	90.38 ± 12.2 aA
Time to first E2 (min)	111.94 ± 16.45 aB	100.22 ± 14.10 aB	77.70 ± 10.39 aB	183.99 ± 20.58 aA	154.64 ± 14.64 bA	197.22 ± 22.21 aA
Sustain phloem ingestion (E2 >10 min)	2.93 ± 0.32 a	3.08 ± 0.40 a	2.52 ± 0.26 a	-	-	-

Means ± SE followed by the different lowercase letters indicate significant differences among the three temperature treatments within the same aphid species using the Bonferroni method at a 5% significance level, while different uppercase letters indicate significant differences between *Ap. gossypii* and *Ac. gossypii* within the same temperature treatment using Student’s t-test at a 5% significance level. EPG waveforms are E1 = salivary secretion; E2: phloem sap ingestion; single E1 = salivary secretion in phloem not immediately following or preceding by the phloem ingestion. The numbers in parentheses are the numbers of each temperature treatment.

## Data Availability

All data analyzed in this study are included in this article.

## Supplementary Materials

The following are available online at https://www.mdpi.com/article/10.3390/insects12060565/s1. Figure S1: A direct current electrical penetration graph amplifier system with Faraday cage.
