# Supplementary figures and images for: Impact of Temperature on Survival Rate, Fecundity, and Feeding Behavior of Two Aphids, Aphis gossypii and Acyrthosiphon gossypii, When Reared on Cotton
Source: Insects. 2021 Jun 21;12(6):565. doi: 10.3390/insects12060565 (PMC8235302; doi:10.3390/insects12060565)

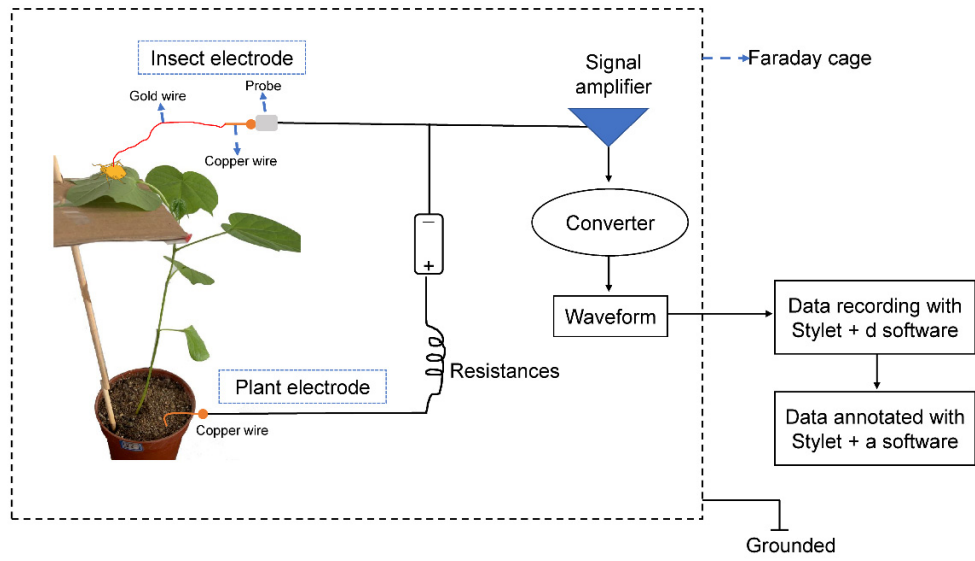

Figure S1 A direct current electrical penetration graph amplifier system with Faraday cage

Supplement: Supplementary file 1 [file insects-12-00565-s001.zip › insects-1220746-SI.pdf]
